# The Nursing Institute

**Published:** 1907-10-05

**Authors:** 


### NURSING ADMINISTRATION.

### THE NURSING INSTITUTE.

(*Continued from page* 673.)

### THE WAY TO SUCCESS.

THE successful nursing institute renders high service to the public, to the medical profession, and to nurses in general. We have shown cause for the opinion that it has a better opportunity of fulfilling all its various responsibilities under the management of an influential committee than when undertaken by a private person, but none the less the position of the superintendent under either system of management is of so responsible a character that it is mainly through her action that the undertaking will prosper or dwindle. The committee may relieve her of much anxiety, save her possibly from mistakes of judgment, and guarantee her from all financial risk, but it cannot do her work. It is on the superintendent even more than on the staff that the doctors will rely, and if she is efficient, it will seem that all the nurses are efficient too. This is no mere fancy. A clever superintendent knows to an ounce exactly what each member of the staff can be called on to do. She has an instinct akin to genius for placing square pegs in square holes, and round pegs in round holes. She believes in her nurses, and when the patient's anxious friends overwhelm her with details of the paragon who alone could ever be admitted within their walls, the superintendent who knows her business has always at command exactly the very nurse who meets the description. It is not that she embroiders the characteristics of her staff, but she is herself persuaded so forcibly that the very qualifications required are latent in her nurses that they positively appear at will. She gives just that sense of moral support which to the timid woman always expecting a contretemps is life-giving. And she has equally the art of dealing cleverly with the stupid members of the staff, and what staff was ever without these well-meaning creatures who at every possible opportunity will do the wrong thing? She can send them where their weak submission to circumstances will count for a virtue, and is often gratified by the successes of her apparent failures. This art, then, of dealing with human nature, the talent called "administrative" for making the best of material, is the great feature of the able superintendent. It goes with a keen eye for character in the choice of the staff, and when combined with a spirit of absolute justice is priceless.

The recurrent difficulty in the supply of nurses is the spasmodic character of the demand. All the problems which vex nurses and puzzle the public in the matter of fees are connected with the fluctuations of disease which extend, oddly enough, even to surgical practice. Ought the nurses to receive a regular salary and be independent of these seasonal fluctuations, or should they receive their own earnings less a percentage for expenses in connection with the agency and take their chance of what work they can get? It is impossible to lay down any hard and fast rule about this matter. In London where the supply of work is fairly constant there can be no question on the advantage to the nurses of the percentage system, tested, as it has been, now for many years by large co-operations in which, perhaps, the highest level of pay ever reached in private nursing is constantly maintained. Nursing in London, cannot, it is true, be quoted as typical of the whole of England. It goes without saying, however, that the more closely the size of the staff can be adjusted to the demand for nurses, the more satisfactory will be the result to nurses and to the institute. Under the co-operation system, the danger is for too many nurses to be engaged. Less risk is involved in this way for the heads of the undertaking than when the supply runs short, and nurses frequently suffer great hardships under a nominally liberal rate of remuneration because work is scarce. Hence, in places where the demand is somewhat intermittent, it is, in our judgment, fairer to engage a regular staff at a salary and let the risk fall on the institute, which has many ways of providing against absolute loss, rather than on individuals. The rotation system which takes the names of nurses in order and offers cases under a rule of precedence to her whose name next occurs in order, is excellent, and tends to promote a sense of being fairly used by all who are employed. It cannot, of course, be adhered to with mechanical regularity or the choice of the superintendent would be nullified, but since cases fall as a rule under well-understood headings, surgical, medical, infectious, mental, obstetric, etc., it is possible to follow an orderly arrangement by which each gets her fair share of work. It is the neglect of this principle which causes so much dissatisfaction in small co-operations unable to secure regular work for all their members.

The question of reduced fees is always present to the superintendent. However anxious she may be to maintain a good standard of payment, there are always cases in which to adhere to a hard and fast rule means absolute rejection. Here the tactful superintendent is generally successful. She cannot press her nurses to accept cases at reduced fees. But when she is fully convinced of the need for a reduction she may often place a case by setting the circumstances before disengaged members of the staff and letting them volunteer. This is specially true of chronic cases, which are readily accepted by nurses who dislike constant change, and the demand for good chronic nurses is so steady that the superintendent does well to keep herself in touch with responsible nurses whose references she has taken up, although for various reasons they are not members of the regular staff.

Some effort ought to be made to induce nurses who are attracted to a nursing institute to provide against sickness and old age. Affiliation to the Royal National Pension Fund for Nurses and inducements offered to nurses who join are among the first duties of the nursing institute to its staff.

